# Reproducibility of Brain Responses: High for Speech Perception, Low for Reading Difficulties

**DOI:** 10.1038/s41598-019-41992-7

**Published:** 2019-06-11

**Authors:** Paavo H. T. Leppänen, Dénes Tóth, Ferenc Honbolygó, Kaisa Lohvansuu, Jarmo A. Hämäläinen, NEURODYS WP7 group, Jürgen Bartling, Jennifer Bruder, Yves Chaix, Stephanie Iannuzzi, Rodolphe Nenert, Nina Neuhoff, Silke Streiftau, Annika Tanskanen, Jyrki Tuomainen, Jean-Francois Demonet, Gerd Schulte-Körne, Valéria Csépe

**Affiliations:** 1 Centre for Interdisciplinary Brain Research, Department of Psychology, P.O. Box 35, 40014 University of Jyväskylä, Jyväskylä, Finland; 2 Brain Imaging Centre, Research Centre for Natural Sciences, Hungarian Academy of Sciences, 1519 Budapest, P.O. Box 286, Hungary; 3 Université de Toulouse, UPS, Imagerie cérébrale et handicaps neurologiques UMR 825; CHU Purpan, Place du Dr Baylac, F-31059 Toulouse Cedex 9, France; 4 Leenaards Memory Center, Département Neurosciences Cliniques, Centre Hospitalier Universitaire Vaudois (CHUV) & University of Lausanne, Rue du Bugnon 46, CH-1011 Lausanne, Switzerland; 5 Department of Child and Adolescent Psychiatry, Psychosomatics, and Psychotherapy, Ludwig-Maximilians-Universität, Nußbaumstr 5a, 80336 Munich, Germany; 6 Inserm, Imagerie cérébrale et handicaps neurologiques UMR 825, F-31059 Toulouse, France; 7 Language and Cognition, University College London, Gower Street, London, WC1E 6BT UK

**Keywords:** Neurodevelopmental disorders, Risk factors

## Abstract

Neuroscience findings have recently received critique on the lack of replications. To examine the reproducibility of brain indices of speech sound discrimination and their role in dyslexia, a specific reading difficulty, brain event-related potentials using EEG were measured using the same cross-linguistic passive oddball paradigm in about 200 dyslexics and 200 typically reading 8–12-year-old children from four countries with different native languages. Brain responses indexing speech and non-speech sound discrimination were extremely reproducible, supporting the validity and reliability of cognitive neuroscience methods. Significant differences between typical and dyslexic readers were found when examined separately in different country and language samples. However, reading group differences occurred at different time windows and for different stimulus types between the four countries. This finding draws attention to the limited generalizability of atypical brain response findings in children with dyslexia across language environments and raises questions about a common neurobiological factor for dyslexia. Our results thus show the robustness of neuroscience methods in general while highlighting the need for multi-sample studies in the brain research of language disorders.

## Introduction

The importance of reproducibility is acknowledged by most researchers^1^. However, an increasing number of studies currently report single results, which can be false positive findings unless they are replicated^2^. This also applies to the past two decades of research uncovering causes of developmental dyslexia, a disorder leading to dysfluent reading in about 3–7% of the population^3^. A large body of empirical evidence (for review, see^3^) suggests that dyslexia is characterized by atypical phonological processing, which is manifested as problems in identifying and manipulating the elements of speech (phonemes and syllables). At the same time, the phonological processing deficit may be the manifestation of different kinds of underlying problems, which are still debated. These include basic auditory processing deficits^4–6^, weakly formed speech sound representations or access problems to these representations^3,7^, and how universal these deficits are across languages^8^. One of the most prominent reasons for the ongoing debate is the failure to replicate findings, partly due to using different study designs and stimulus conditions and lack of statistical power, as well as different language environments of the participants.

To investigate the basic auditory and speech perception problems and the potential of brain responses as a neurobiological marker of dyslexia, we used the event-related brain potentials (ERP) focusing on the mismatch negativity (MMN)^9^ and the late discriminative negativity (LDN)^10^ both widely used in dyslexia research. MMN indicates discrimination and detection of change in an auditory stream^9^, while LDN is related to further discriminative processing^10^. Atypical MMN^11–18^ or LDN^13,19^ in dyslexia are independently reported by different research groups from several language environments, for example Chinese, English, Finnish, German, Hungarian (for reviews, see^20,21^). This might seem to imply that the altered MMN in dyslexia is a universal phenomenon. However, there are also findings for other atypical brain responses such as M210^22^, and the atypical MMN in individuals with dyslexia have been sometimes found exclusively for speech sounds and sometimes for non-speech sounds^23–25^. This indicates the possibility that experimental designs, study samples or stability of the ERP responses can lead to slightly different results in the group comparisons. Therefore, we need multi-center studies to clarify these conflicting findings.

Here we report on a unique attempt of four research groups in four countries to test the consistency of atypical speech processing in developmental dyslexia in an ERP study with a large school-aged sample of ca. 400 children. To address the problem of inconsistent results we applied a strictly controlled cross-linguistic design. We (1) selected dyslexic and control participants according to the same procedure, using nationally normed behavioral tests for reading fluency, (2) used the same stimulus set and experimental procedure, (3) applied the same data recording parameters and processing steps to extract averaged ERPs, and (4) followed a systematic and automatized analysis procedure using R packages^26^ (see Materials and methods).

**Figure 1:**
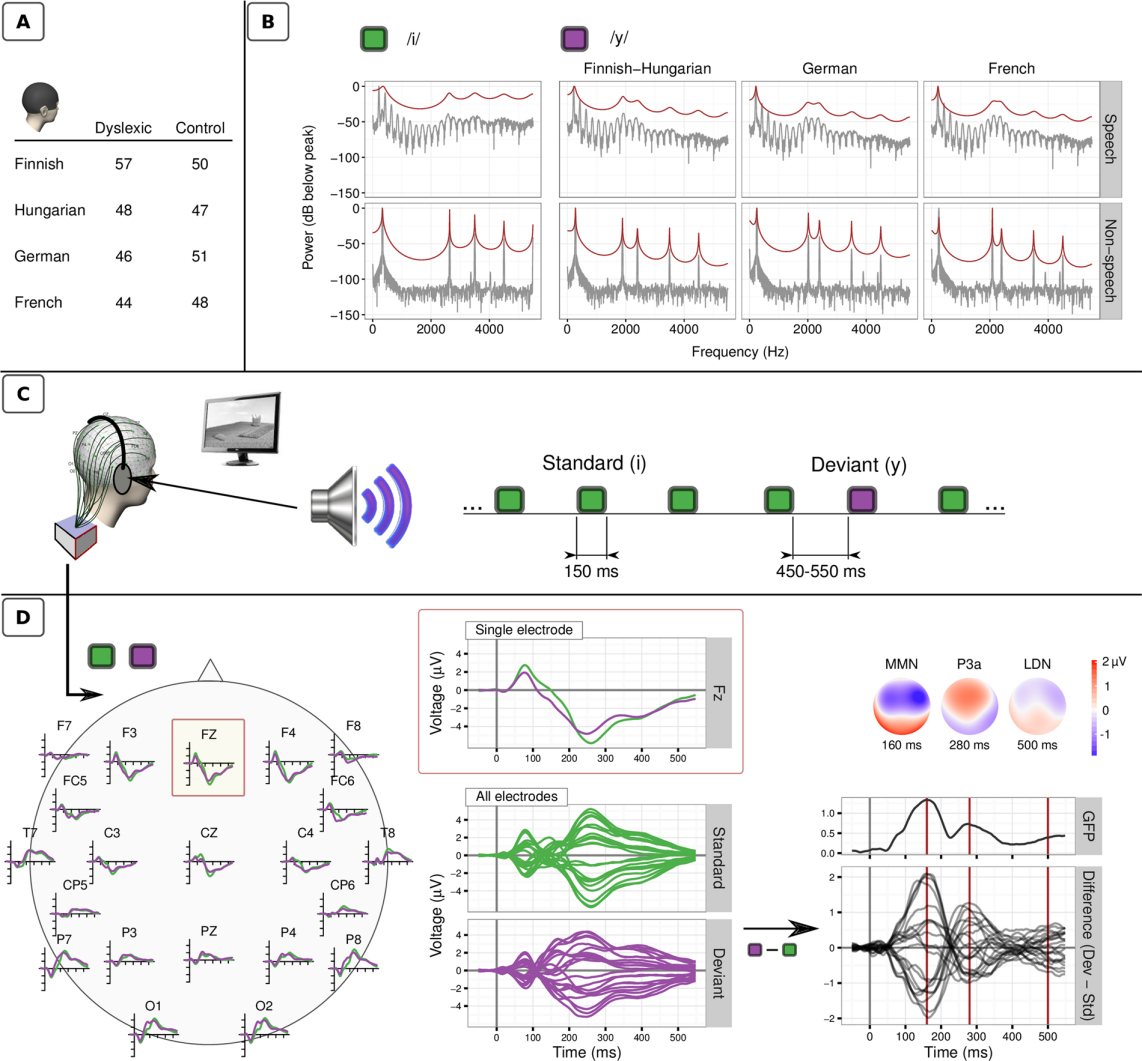
Outline of the experiment. (**A**) Sample sizes: Large samples of dyslexic and control children were recruited in four countries according to common inclusion criteria and normed screening tests. **(B)** Stimuli: Based on a series of behavioral studies, three prototypes of the speech sound/y/(one for each language group: Finnish-Hungarian, German, French) and one common prototype of the speech sound/i/were selected. Non-speech variants of the vowels were synthesized by omitting vocal tract resonances. They served as a control condition for the acoustic differences between stimuli. **(C)** Experimental protocol: The stimuli were presented in a passive oddball paradigm: the sequence of standard/i/sounds were occasionally interrupted by the language-specific/y/(the deviant stimulus), delivered in six blocks (2 [speech/non-speech] X 3 [Finnish-Hungarian, German, French deviant]). The participants watched a silent movie without any explicit task. **(D)** An example of grand averaged ERP waveforms and scalp distributions in the German control sample in response to the standard/i/and deviant German/y/sounds. A common subset of 21 electrodes was included in the analyses. For each electrode and each sampling point, amplitudes to the deviants and standards were compared, and the Global Field Power (=standard deviation) of the channel-wise differences were computed. The three peaks representing the ERP components of interest are highlighted and the corresponding scalp topographies are plotted. The overall response pattern was similar in all samples.

A cross-linguistic passive oddball paradigm using/y/-/i/vowel contrasts was created for 8–12-year-old children (N = 391, Tables 1 and 2, Fig. 1) speaking one of four different languages (Finnish, Hungarian, German, and French, in decreasing order of orthographic consistency^27^). Three deviant types were used based on Finnish/Hungarian, German and French phonology (Fig. 1). In addition, an experiment with complex non-speech sounds was conducted to control whether reproducibility and dyslexia-related effects are speech specific. As the main analysis method, topographic analysis of variance (TANOVA, described in^28^), taking into account both the amplitude and topography of ERP activity, was applied for each time point across the averaged responses (see Materials and methods for details). As there were no systematic differences in the speech MMN and LDN responses between the three/y/-vowels in any of the samples (see Supplementary Figs 5 and 6), the different/y/-vowel responses were averaged.

**Table 1:** Sample sizes, gender, and handedness.

Sample	Group	Total	M	F	R	L	A
Finnish	Control	50	25	25	45	3	2
	Dyslexic	57	36	21	51	4	2
Hungarian	Control	47	24	23	40	7	0
	Dyslexic	48	27	21	44	4	0
German	Control	51	27	24	44	6	1
	Dyslexic	46	24	22	40	6	0
French	Control	48	24	24	39	9	0
	Dyslexic	44	34	10	40	3	1

Note. M = Male, F = Female, R = right handed, L = left handed, A = ambidextrous. The handedness was tested with Edinburgh inventory^45^ in Finland and France, the Annett Hand Preference Questionnaire^46^ in Hungary, and the Leistungs-Dominanztest^47^ in Germany.

**Table 2:** Age, reading, phonological processing, and general cognitive abilities of the participants as well as group differences in these measures.

Sample	Variable^a^	Group	Valid N	Mean	SD	Min	Max	t^b^
Finnish	Age	Cntrl	50	10.26	0.43	9.55	11.05	1.35
		Dysl	57	10.12	0.64	8.42	11.60	
	Read	Cntrl	50	0.25	0.73	−0.67	1.33	17.43***
		Dysl	57	−1.80	0.42	−3.00	−1.33	
	PhonDel	Cntrl	50	0.02	0.95	−1.84	1.81	3.43***
		Dysl	57	−0.69	1.18	−2.85	1.81	
	Blocks	Cntrl	50	10.86	2.38	7.00	16.00	0.69
		Dysl	57	10.56	2.09	6.00	15.00	
	Similarities	Cntrl	50	12.54	3.48	6.00	19.00	2.71**
		Dysl	57	10.93	2.52	6.00	18.00	
Hungarian	Age	Cntrl	47	9.91	0.88	8.36	11.76	−0.07
		Dysl	48	9.92	0.99	8.23	12.04	
	Read	Cntrl	47	0.59	0.77	−0.79	2.41	18.66***
		Dysl	48	−1.81	0.44	−3.09	−1.25	
	PhonDel	Cntrl	47	0.22	0.85	−1.53	2.21	8.66***
		Dysl	48	−1.39	0.96	−3.81	0.62	
	Blocks	Cntrl	46	12.61	2.74	8.00	18.00	1.92
		Dysl	48	11.46	3.07	7.00	19.00	
	Similarities	Cntrl	46	13.00	2.78	8.00	18.00	3.72***
		Dysl	48	10.77	3.03	6.00	19.00	
German	Age	Cntrl	51	9.76	0.69	8.74	11.41	1.60
		Dysl	46	9.53	0.75	8.35	11.70	
	Read	Cntrl	51	0.77	0.76	−0.60	3.20	20.54***
		Dysl	46	−1.98	0.55	−3.20	−1.30	
	PhonDel	Cntrl	50	0.02	0.97	−1.78	2.98	6.04***
		Dysl	46	−1.34	1.20	−4.34	1.15	
	Blocks	Cntrl	51	10.78	2.02	6.00	14.00	0.75
		Dysl	46	10.43	2.50	6.00	16.00	
	Similarities	Cntrl	51	13.61	2.29	8.00	19.00	3.43***
		Dysl	46	11.76	2.94	4.00	19.00	
French	Age	Cntrl	48	10.09	1.01	8.21	11.85	0.01
		Dysl	44	10.09	1.10	8.09	12.03	
	Read	Cntrl	48	1.33	1.47	−0.79	6.89	13.98***
		Dysl	44	−1.72	0.35	−2.56	−1.25	
	PhonDel	Cntrl	45	0.12	0.97	−2.85	2.46	6.43***
		Dysl	39	−1.28	1.01	−3.78	0.75	
	Blocks	Cntrl	48	11.02	2.62	7.00	18.00	−0.05
		Dysl	44	11.05	2.11	7.00	15.00	
	Similarities	Cntrl	48	12.81	2.40	9.00	19.00	2.85**
		Dysl	44	11.34	2.54	5.00	16.00	

Note. ^a^Age = chronological age in years; Read = word reading (z-scores); PhonDel = phoneme deletion (z-scores); Blocks = Block Design (WISC); Similar = Similarities (WISC). ^b^Group comparisons: Separate t-tests were conducted for all variables in each national sample; stars denote significant (uncorrected) differences between the Control (Cntrl) and Dyslexic (Dysl) groups: ***p < 0.001, **p < 0.01, *p < 0.05.

## Results

### Reproducibility of brain responses related to change detection

**Figure 2:**
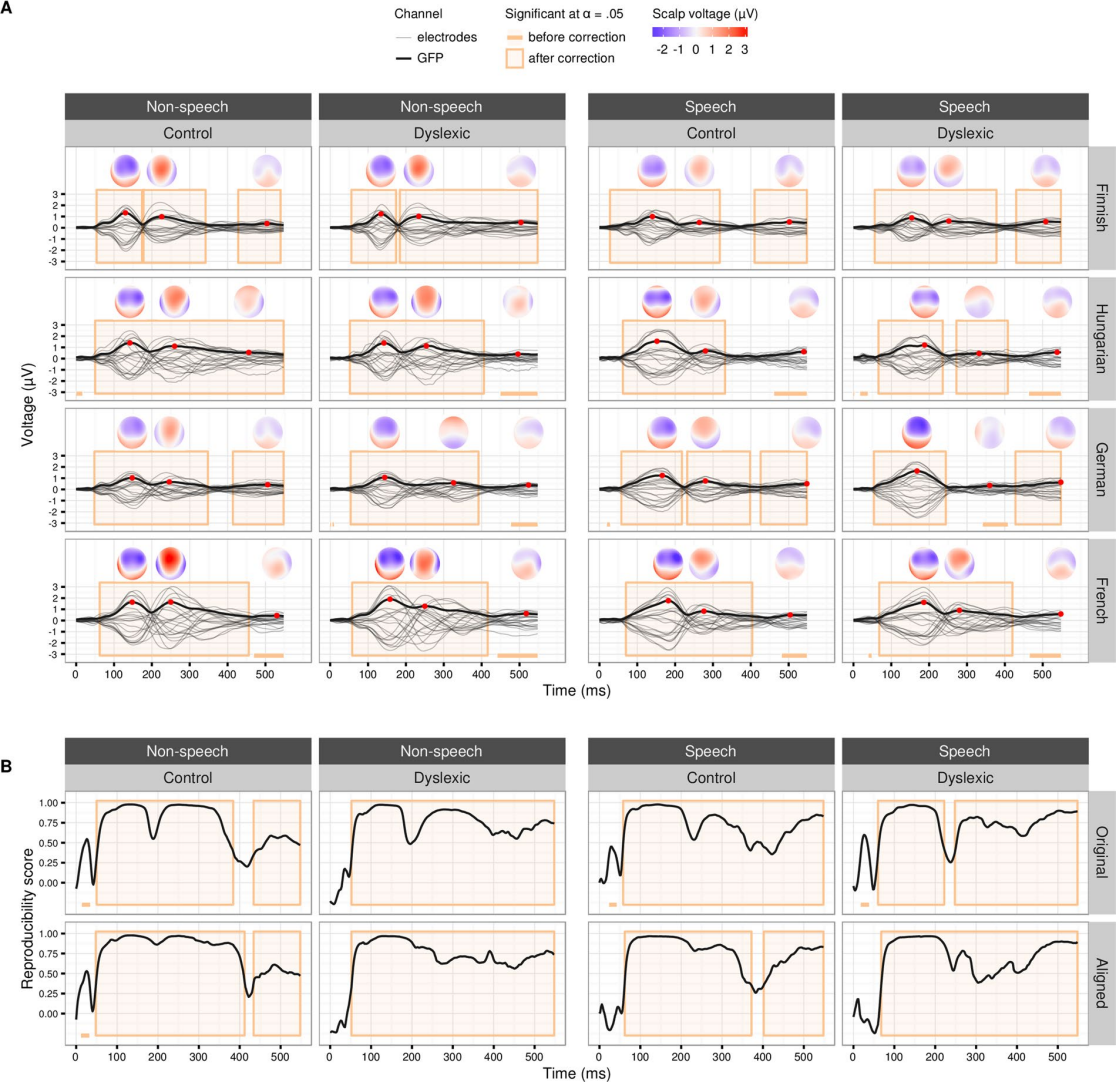
The effect of the stimulus type and its reproducibility. (**A)** Difference waves (the deviant responses minus the standard responses, electrodes in grey and global field power, GFP, in black) and topographies (from the time points marked with the red dots) for the non-speech (left) and speech (right) stimuli for each national sample and reading group. **(B)** Reproducibility scores [RS] between national samples for each time point in the whole epoch in the non-speech (left) and speech (right) conditions for the reading groups separately.

The MMN topography at 100–200 ms showed the distinctive features of a typical MMN component in both speech and non-speech conditions (Fig. 2A, Supplementary Figs 1 and 2). The MMN was followed by a P3a component thought to reflect involuntary attention switch to deviating stimuli in a sound stream^29^. Late discriminative negativity (LDN) emerged between ca. 350 ms and the end of the epoch. The three components were present as evidenced by significantly larger responses to the deviant than the standard stimuli for the speech and non-speech conditions for most of the samples: **Speech** MMN (*P* ≤ 0.00001) with all effects being persistent according to the minimum duration criterion ([MDC]: *P*_*MDC*_ ≤ [0.001, 0.01]) with very large effect sizes (η^2^_G_ = [0.63, 0.78]), P3a (*P* ≤ [0.00001, 0.0033], η^2^_G_ = [0.27, 0.52]) being persistent in all samples (*P*_*MDC*_ ≤ [0.001, 0.05]) except in the German dyslexic sample, and LDN (*P* ≤ [0.00001, 0.0036], η^2^_G_ = [0.23,0.42]) being persistent only in the Finnish and German samples (*P*_*MDC*_ ≤ 0.05); **non-speech** MMN (*P* ≤ 0.00001, *P*_*MDC*_ ≤ [0.001, 0.05], η^2^_G_ = [0.58, 0.72]), P3a (*P* ≤ 0.00001, *P*_*MDC*_ ≤ [0.001, 0.01], η^2^_G_ = [0.39,0.80]), and LDN (*P* ≤ [0.00001, 0.0054], η^2^_G_ = [0.25, 0.40], being persistent in all but the French control and dyslexic, and German and Hungarian dyslexic samples.

As we were interested in the reproducibility of the results between different countries, we introduced a quantitative measure, Reproducibility score (RS), to evaluate the scalp topography similarity of the stimulus type effect between countries (see Materials and methods) with the value of 1 showing perfect correlation of the effect maps between the samples and value of 0 showing completely non-correlating effect maps between the samples (Fig. 2B).

The effect of stimulus type, indicating a difference between the processing of standard and deviant stimuli, was highly reproducible in all conditions within the dyslexia group and within the control group with and without time-alignment for MMN and P3a (MMN: *RS*_*max*_ = [0.97, 0.98], P ≤ 0.0001, *P*_*MDC*_ ≤ [0.001, 0.01], P3a: *RS*_*max*_ = [0.71, 0.98], *P* ≤ [0.0001, 0.0025], *P*_*MDC*_ ≤ [0.001, 0.01], with and without time-alignment, respectively) (see Fig. 2B). For the LDN response, the reproducibility showed more variance (LDN: *RS*_*max*_ = [0.59, 0.90], P ≤ [0.0001, 0.0021], *P*_*MDC*_ ≤ [0.01, 0.05]), being lowest in the non-speech condition in the control group. Here we, for the first time, tested statistically the reproducibility of the MMN component recorded in four different laboratories and national samples and found it to be extremely reproducible and consistent across language environments.

### Reproducibility of the brain responses between the reading groups

**Figure 3:**
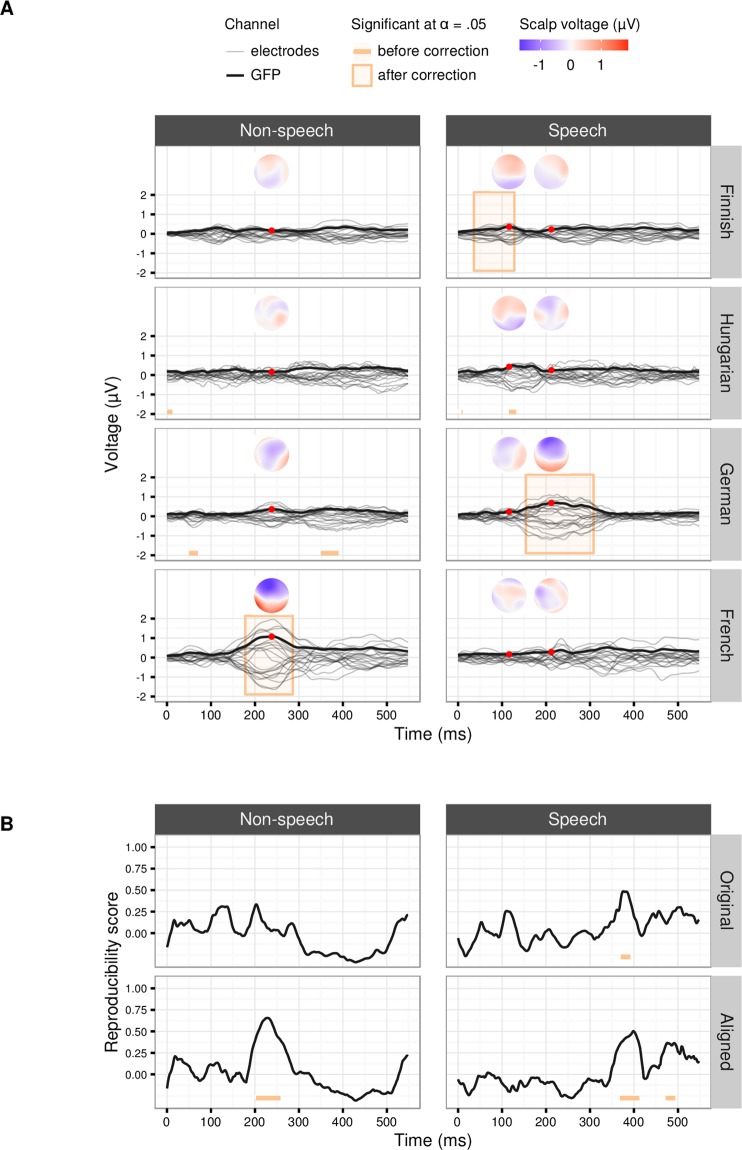
The effect of the reading group on deviant-standard difference and its reproducibility. (**A**) Reading group difference waves (the responses of dyslexic minus those of control participants) of the deviant-standard difference waves (electrodes in grey and global field power, GFP, in black) and topographies (from the time points marked with the red dots) for the non-speech (left) and speech (right) stimuli for each national sample. The time ranges with statistically significant differences between the groups are highlighted. (**B**) Reproducibility scores for group differences between the national samples in non-speech (left) and speech (right) conditions.

In the next step, we examined whether the reading group membership modulates the difference between the deviant and standard responses, and whether these group differences are reproducible. The TANOVAs showed significant reading group by stimulus type interactions in all but one national sample, albeit divergently in the different countries as seen in Fig. 3. These results clearly indicate that discriminative auditory brain responses do differ between typical and dyslexic readers at MMN and P3a time windows, but at different time windows and even in different conditions between national samples. For speech stimuli, German dyslexic children produced larger MMN and smaller P3a responses than control children, the differences being persistently significant between ca. 150–310 ms (*P* ≤ 0.00004, *P*_*MDC*_ ≤ 0.01, η^2^_G_ = 0.026). On the contrary, Finnish dyslexics had smaller responses at ca. 35–130 ms (*P* ≤ 0.00456, *P*_*MDC*_ ≤ 0.05, η^2^_G_ = 0.012), where the later portion of the time window matched with the occurrence of overlapping N1 and MMN components. A similar, but statistically not persistent effect appeared in the Hungarian sample between 70–170 ms (*P* ≤ 0.004011, *P*_*MDC*_ > 0.05, η^2^_G_ = 0.012). For the non-speech stimuli, French dyslexic children had a larger MMN but smaller P3a than control children at ca. 170–320 ms (*P* ≤ 0.0001, *P*_*MDC*_ ≤ 0.05, η^2^_G_ = 0.048).

This inconsistency of the group differences between the national samples was also captured by the reproducibility measure, which was low (*RS*_*max*_ = 0.66 and 0.50 in the non-speech and speech conditions, respectively, for the time-aligned responses) and not statistically persistent (*P* ≤ 0.0063 and 0.0132, respectively *P*_*MDC*_ > 0.05) (Fig. 3B).

## Discussion

Our results show the robustness of change detection related responses in the study of auditory and speech perception while highlighting the need for multi-sample studies in the brain research of language disorders. The results draw attention to the limited generalizability of atypical MMN, P3a and LDN findings in children with dyslexia from different language environments.

Our study with four national samples shows results, which could have been interpreted, if they had been found in separate studies, as an indicator of a general impairment in basic auditory (in the French sample) or speech processing (for example in the Finnish sample) in dyslexic readers as it has previously been suggested in several studies (e.g.^11,19^). In the French sample, a diminished P3a for the non-speech sounds was found in children with dyslexia compared to controls which could have indicated a domain general processing deficit manifested in automatic attention switching mechanisms^29^. However, none of the other national samples showed any group differences for the non-speech sounds leading to the rejection of the conclusion of impaired attention switching as a universal and general mechanism related to dyslexia. While P3a was also diminished in the German dyslexia sample for the speech sounds, P3a was present in dyslexic readers in the other two national samples, Finnish and Hungarian, for both the non-speech and speech stimuli. Furthermore, the individuals with dyslexia in the German sample showed an enhanced MMN response for the speech stimuli, whereas the Finnish and Hungarian dyslexia groups showed diminished speech MMN responses compared to controls. The Finnish and Hungarian results would be in line with many previous studies showing smaller MMN responses in individuals with dyslexia and therefore implicating problems in phonological processing (e.g.^11,13–17^). The results from the German sample, however, argue against diminished MMN for speech as a universal marker for dyslexia as in that sample an enhanced MMN was linked with dyslexia. This is further confirmed by the lack of MMN differences for speech in the French sample.

The lack of group differences for non-speech MMN in all national samples in our study is in line, though, with previous findings for the rather large frequency difference between the standard and the deviant tones as was the case in our study for complex non-speech sounds^21^. It has been suggested that differences in frequency discrimination ability or MMN responses between individuals with dyslexia and those with typical reading skills emerge only for small frequency changes (<10% difference)^11,21,30^.

Given that our results are based on large sample sizes of ca. 100 children in each national sample, with carefully matched and rigorously controlled experimental design, data acquisition and analyses, they likely represent real – albeit small – brain response differences between typical and dyslexic readers. It should also be noted, that in the examination of the signal-to-noise ratio (SNR, Supplementary Fig. 8) we could not identify any systematic differences between the national samples, reading groups or conditions. Therefore, we can conclude that technical differences in data collection or pre-processing do not systematically affect the current results. The moderate group differences in this study could thus be, in part, explained by the relatively robust stimulus contrasts (/y/vs./i/, and the equivalent non-speech contrast). This implies, on the other hand, that significant group differences show rather gross atypical processing of speech material in dyslexic readers, but not in all language or national environments.

Our results are not, however, directly comparable with previous speech MMN studies, where the differences between controls and dyslexic readers have been typically investigated with consonant-vowel syllables. For example, in German speaking samples group differences have often emerged at the LDN time window (e.g.^13,19^), but also at the MMN time window (e.g.^13,14^). The previous results at the MMN time window have shown a diminished response^14^ or enhanced positive response^13^, both of which are at odds with the current findings using only vowel stimuli.

Previous results and our results thus suggest that the neural level risk factors for dyslexia involve variations that are not universal, but are affected by different experimental variables, genetic profiles and environmental factors such as the phonological system of the language. Although the different/y/-vowel exemplars in our study did not produce statistically observed differences between MMN responses to these exemplars (see Supplementary Results), it is still possible that differences between the phonological systems of different languages were reflected in the top-down effects of long-term representations on speech perception. This option is indirectly supported by the behavioral preference ratings for the/y/-vowel carried out for adult participants in producing the current stimuli (see Experimental design/Stimuli). These preference ratings show that the phonemic maps for different languages are different for this vowel. The Finnish and Hungarian adults preferred the same/y/-exemplar and the German and French adults, on the other hand, preferred different/y/-vowels as the best exemplar of their respective languages. We speculatively suggest, as one possibility, that the phonological language background could also play a role in the patterns of ERP group differences in different national samples found here between children with reading problems and those with typical reading skills. This possibility should be examined in the future studies.

Our findings thus clearly show that caution is needed in interpreting cognitive neuroscience results solely based on one language or country. The results also encourage examination of individual differences going beyond the current reporting of descriptive group level differences in brain responses and towards uncovering the exact neural mechanisms of language related disorders.

## Materials and Methods

### Participants

Altogether 409 children participated in Finland, France, Germany, and Hungary in a cross-linguistic brain event-related potential (ERP) study as a part of the European Sixth Framework Programme NeuroDys-project (Dyslexia genes and neurobiological pathways)^31^ (Tables 1 and 2). The final sample with good quality EEG data consisted of 391 children (195 dyslexics, 196 controls; 221 males; age range: 8–12, mean: 9.97, SD: 0.85).

The reading level was determined using language-appropriate standardized word reading tests (Finland: Lukilasse^32^; Hungary: 3DM-H^33^; Germany: SLRT II^34^; France Odedys^35^). Both accuracy and speed were assessed and converted into a composite word reading fluency measure (number of correctly read words per minute). To ensure inclusion of only average (or above average) readers in our control sample, children belonging to the control group were required to be at least or above −0.85 standard deviations from the average in grade-appropriate norms. Dyslexic children were required to be 1.25 or more standard deviations below the average.

All participants had an IQ of at least 85 as measured with non-verbal (Block design) and verbal (Similarities) subscales of the Wechsler Intelligence Scale for Children, third or fourth edition^36,37^. Participants with dysphasia, language impairments (including specific language impairment, SLI), attention deficit hyperactivity disorder (ADHD), neurological illness, head injury, medication for a psychiatric disorder, epilepsy, or uncorrected poor eyesight were excluded. The hearing level measured with audiometer was required to be normal. Further exclusionary criteria were an exposure to Finnish, French, German, or Hungarian as a foreign language, including one of the parents speaking these languages to the participant, attending kindergarten or school using these languages or living in a foreign country speaking one of these languages for more than a year. In all four countries, the study received approval of the relevant institutional and licensing ethical committees: The University of Jyväskylä Ethical Committee, Jyväskylä, Finland; United Ethical Review Committee for Research in Psychology, Budapest, Hungary; Comite consultatif pour la protection des personnes dans la recherche biomédicale (CCPPRB), Toulouse 1, France; and Ethikkommission der Medizinischen Fakultät der Ludwig-Maximilians-University, Munich, Germany. Informed consent was obtained from all parents prior to their child’s inclusion in the study and the study was conducted according to the Declaration of Helsinki. For detailed sample sizes and participant characteristics, see Tables 1 and 2.

### Experimental design

#### Stimuli

The stimuli were synthesized using Praat software v. 4.5^38^. The stimulus set consisted of three synthetic/y/vowels and one/i/vowel, and their complex non-linguistic counterparts consisting of five sinewave tones located at frequencies corresponding to the lowest five formants of the synthetic vowels. The stimulus duration was 150 ms for each, and a linear 10 ms onset and 15 ms offset ramp was applied. Originally, a set of 135 synthetic vowels were created for a behavioral experiment for stimulus selection for subsequent ERP studies. The vowels covered the formant space of the/y/phoneme and surrounding front high and front mid-high vowels of Finnish, French, German, and Hungarian (120 designated as/y/, and 15 catch trials designated as/i/,/e/, and/oe/). The phoneme boundaries and goodness (acceptability) of the vowel stimuli were evaluated by 81 normal adults (Finland, N = 20; France, N = 20; Germany, N = 21, and Hungary, N = 20). In each country, one vowel identified as/y/with at least 85% accuracy and receiving the highest goodness rating was selected for the stimulus set to be used in the behavioral discrimination and ERP experiments. As the Finnish and Hungarian listeners preferred the same vowel, only three/y/vowels were chosen: a Finnish-Hungarian, a French, and a German/y/for the subsequent experiments. One common “euro”-/i/vowel was synthesized representing the average formant frequencies of a “typical”/i/vowel of all the four languages. For the formant frequencies of the vowels, see Table 3.

**Table 3:** The acoustical properties (the first three formants) of the vowel stimuli, and the Euclidean distance of the F1 and F2 values of/y/sounds from/i/. All values are given in Hz.

	*F1*	*F2*	*F3*	*Euclidean distance from/i/*
“euro”/i/	335	2638	3500	
Finnish-Hungarian/y/	274	1886	2400	755
German/y/	250	2018	2400	626
French/y/	250	2086	2400	559

In addition to the formant frequencies shown in Table 3, for the/i/vowel 6 formants were used at 4500, 5500, 6500, 7500, 8500, and 9500 Hz. For all/y/vowels, seven additional formants were used at 3500, 4500, 5500, 6500, 7500, 8500, and 9500 Hz. The glottal source was created by converting the pitch and timing information to a glottal source signal (0.1% noise was added to make the signal sound more natural). The duration of the source signal was 150 ms and the pitch fell linearly from 230 Hz at the onset to 200 Hz at the offset (mean pitch 5 Hz). The source was filtered with a vocal tract model containing information about the frequencies and bandwidths of the 10 lowest formants (i.e., vocal tract resonances). Female pitch characteristics were used.

The corresponding non-speech stimuli were also created by synthesizing five separate sine wave tones at the frequencies corresponding to the first five formant peaks used in the synthesis parameters without using the glottal source. The amplitudes of the sine wave tones were matched according to values obtained by directly measuring the formant amplitudes of the selected synthesized vowels. Finally, all five sine tones were combined to create a complex tone.

For the spectral characteristics of the speech and non-speech stimuli, see also Fig. 1B.

#### Procedure

The stimuli were presented in two separate, speech and non-speech, passive oddball conditions; the non-speech condition was always presented first to prevent participants from perceiving the non-speech stimuli as speech. The deviant stimuli were Finnish-Hungarian, German, and French/y/s presented in three separate blocks, while the standard stimulus was always the common ‘euro’/i/in each block. The block order was counter-balanced within each condition. Altogether, 717 stimuli were presented in each block, with 18/82% proportion of the deviant and standard stimuli, respectively. The average stimulus onset asynchrony varied randomly between 600–700 ms (average 650 ms). Also, the number of the standard stimuli varied pseudo-randomly with 3–13 standard stimuli between the two consecutive deviant stimuli. The stimuli were presented binaurally with an intensity of approximately 70 dBA via PX200 Sennheiser headphones. During the experiment, participants watched a silent video and they were instructed not to pay any attention to the stimulus sounds. The experiment was run using E-prime (in Finland and Germany) or Presentation (in France and Hungary) software. The testing session lasted 1.5–2.5 hours depending on the time required for the application of electrodes, pauses, and additional behavioral tasks.

#### EEG recording

In Finland and Germany, the EEG was recorded with Ag-AgCl electrodes using 128-channel (Electric Geodesics Inc.) HydroCel Geodesic Sensor Nets, NetAmps 200 amplifier, and NetStation 4.2.1 software (http://www.egi.com). The EEG was filtered online with a bandpass of 0.1–200 Hz and referred to the Cz-electrode. Electro-oculogram (EOG) was recorded with electrodes located above, below and lateral to the both eyes. All electrode impedances were pursued to be kept below 50 kΩ (the quality of the data were monitored and electrode contact corrected as necessary during the recording). Sampling rate was 500 Hz in all countries.

In France, the EEG was recorded with 32 Ag-AgCl electrodes using Neuroscan amplifier, and Neuroscan 4.2 software, with online bandpass filter of 0.1–100 Hz and referred to the Cz-electrode. EOG was monitored with two electrodes: one placed above the left eye and one placed on the right temple. Impedances for all electrodes were kept below 5 kΩ.

In Hungary, the EEG was recorded with 32 Ag-AgCl electrodes attached to an elastic electrode cap (Easycap, http://www.easycap.de) using BrainVision amplifier system and BrainVision Recorder software (http://www.brainproducts.com). The data were filtered online with a bandpass of 0.1–100 Hz using the Pz-electrode site as a reference. EOG was recorded with four electrodes located above and below and lateral to the eyes. The electrode impedances were kept below 10 kΩ. The electrode impedances in all countries were in accordance with the recommendations for each amplifier type by the manufacturer and standard practices.

Possible differences between country-wise results are unlikely to originate from the technical dissimilarities because 1) the data was normalized before the analyses (see below) and 2) all comparisons were performed separately within each national sample dataset, and 3) the SNR examination did not reveal any systematic sample data differences between the national sample datasets (see Supplementary Fig. 8).

#### Pre-processing of EEG data

The EEG data of each national sample were digitally filtered offline (bandpass: 0.3–30 Hz, 12 dB/octave roll off with a filter type zero-phase). To attenuate electrical noise, the notch filter was set to 50 Hz with the width of 2.0 Hz.

In Finland, the data were pre-processed using BESA 5.3.1 software (www.besa.de). Eye blinks in the data were corrected before averaging with an individual eye blink correction algorithm implemented in BESA using principal component analysis^39^. The channels with multiple artefacts throughout the data were set to bad and omitted from the averaging. EEG epochs with the voltage deflections exceeding ±200 µV were also excluded from the averaging as artefacts. After the averaging, the channels previously set as bad channels were interpolated using a spherical spline interpolation method^40^. In Hungary and Germany pre-processing was done with Brainvision Analyzer. Eye blinks in the data were corrected by using independent component analysis. Artefactual epochs with the absolute voltage exceeding ±150 µV, a gradient exceeding 50 µV on two successive sampling points or a gradient exceeding 150 µV in 200 ms range were excluded from the averaging. In France, the epochs contaminated by eye-movements or other artefacts of non-biological origin producing voltage larger than ±125 μV peak-to-peak were omitted from averaging. The minimum number of epochs accepted for averaging for each stimulus type was 50 in all countries.

The ERPs were obtained by averaging epochs of 600 ms (including a pre-stimulus baseline of 50 ms) separately for the pre-deviant standard stimulus and each deviant stimulus type separately for each participant and condition. Only the responses to the pre-deviant standard stimuli were included in the analysis leading to an equal amount of epochs for the deviant and standard stimuli and thus an equal signal-to-noise ratio. The first 10 trials of each block were excluded from averaging. Deviant-standard difference waves were obtained by subtracting ERPs elicited by the pre-deviant standard stimuli from those elicited by the deviant stimuli.

The problem of different number of electrodes per country (Finland and Germany 128, France and Hungary 32) in slightly different positions was solved by transforming the original averaged data of the Finnish and German groups into the standard 10–10 electrode system by using spherical spline interpolation function in Besa. After that, all electrodes common to all four laboratories were used in the further analyses. These common 21 electrodes were the following: F7, F3, Fz, F4, F8, FC5, FC6, T7, C3, Cz, C4, T8, CP5, CP6, P7, P3, Pz, P4, P8, O1, O2. Finally, the data were re-referenced to the average reference of these common electrodes, and normalized within each national sample dataset before combining into a common dataset. Normalizing was carried out by dividing each data point of the original ERP data by the standard deviation (SD) over all timepoints, channels, stimuli and participants.

### Statistical analysis

All statistical analysess were carried out, using R^26^, on each time point of the individual ERPs between 0 and 548 ms after stimulus onset, separately for the speech and non-speech conditions. We analyzed the effects of one experimental and one participant-level factors: 1) Type: standard (/i/) or deviant (/y/) sound; 2) Group: dyslexic and control readers. The effect of exemplar (the three language exemplars of the/y/sound) were not examined in the main analyses because the exemplars did not produce significant effects on the ERPs (see Supplementary Results). The main analyses were therefore conducted for the responses averaged across the three exemplars.

#### TANOVA analysis

In the first step, we aimed at identifying the time windows with the above factors producing significant effects on the brain responses. We computed Topographic Analysis of Variance (TANOVA^28^) on the ERP maps at each time point in each national sample separately, as follows.

TANOVA is a data-driven, non-parametric method, which is sensitive to differences between both the intensity and/or the topographic distribution of the scalp amplitudes in the different conditions. In TANOVA, a grand average difference wave between the responses to different stimuli (deviant vs. standard) are first calculated for each channel. Then global field power (GFP) of this difference wave is calculated. Third, permutations are then produced (randomly re-assigning labels for the deviant and standard responses of each participant) and the grand average difference wave GFP values at each time point is compared to the distribution derived from the permutations. This gives the p-value for the tested effect. In the present analyses, we calculated the p-values based on 99,999 randomization runs.

In the case of stimulus by group interaction the procedure is the same as above but examined contrast is the group difference of the deviant-standard difference waves (for detail see^28^).

The analysis is conducted for each time point, and therefore adjustment is needed to control for the inflation of Type I error. This was controlled by computing the distribution of the duration of randomly obtained significant periods (defined as the number of consecutive time points which are significant at a given level of alpha) from the permuted data, and comparing the durations of each observed significant period to this distribution (referred to as minimum duration correction, and the time windows which remained significant after correction as persistent effects). In the present study, we tested the following levels of alpha: 0.001, 0.01, 0.05. The analyses were also carried out using Z-scored generalized dissimilarities and these results are reported in the Supplementary material.

To provide complementary information to the TANOVA results, we also applied the more traditional point-by-point ANOVA methodology and calculated the generalized eta squared (η^2^_G_) effect size measure^41^ for each channel separately. The maximum values are indicated in the Result section text.

#### Reproducibility analysis

The national samples can be considered as independent replication samples. The TANOVA analysis reveals the persistent effects for each national sample but does not quantify the between-sample similarity of those effects. To quantify reproducibility, we introduce the Reproducibility Score (RS), inspired by Killeen’s approach^42^, who defined successful replication “as an effect of the same sign as that found in the original experiment” (p. 346). This does not require the significance (*p*-value) of the original effect to be replicated. For ERPs, we considered an effect reproducible if the topography of the effect was similar across the national samples at comparable latencies, irrespectively from the overall intensity or the significance of the effect in each sample. Technically, for each time point, we calculated the effect maps in each sample and computed the correlation matrix using Pearson product-moment correlation. The pairwise correlation coefficients were then averaged to get an overall measure of between-sample similarity: the RS. Note that instead of simple averaging, the correlation coefficients were first normalized by Fisher’s *z* transformation, averaged, and back-transformed to the original scale to obtain a less biased estimate^43^.

One potential caveat with the above procedure is that between-sample latency differences might lead to attenuated RS, even if the topography of the effect is highly similar across the samples. That is, it ignores our less restrictive requirement that effect topographies should be contrasted at comparable, albeit not at the very same latencies. Therefore, we conducted the reproducibility analysis also on time-aligned curves. A time-warping algorithm based on the elastic square-root slope framework^44^ was applied to the GFP of the mean effect curves (e.g., of the channel-wise grand averages of deviant-standard difference waves) to obtain a common warping function for the channels, and the RS was calculated on the warped effect curves.

To test the significance of the RS values, we applied the same randomization strategy and correction method as in the TANOVA analyses (with 9,999 randomization runs). That is, we shuffled the individual ERPs and calculated the effects within national samples, then computed the RS across samples on both the original and time-aligned curves and repeated these steps to create the distribution of the RS under the null. We also adopted the same correction method as described in the TANOVA section to reveal the persistently significant time windows.

We did not include separate peak latency analyses for the ERP responses in this report, because based on the visual inspection of the country-wise grand averages (see Supplementary Fig. 9) there were no clear group effects of latency.

## Supplementary information

Supplementary Materials for Reproducibility of Brain Responses: High for Speech Perception, Low for Reading Difficulties

## Data Availability

All ERP data and codes used in the analysis will be shared upon request. The de-identified data can be requested from the corresponding author along with the R packages for the implemented ERP processing pipeline.
