# Supplementary material for: Reproducibility of Brain Responses: High for Speech Perception, Low for Reading Difficulties
Source: Sci Rep. 2019 Jun 11;9:8487. doi: 10.1038/s41598-019-41992-7 (PMC6560029; doi:10.1038/s41598-019-41992-7)
Supplement: Supplementary file 1 — Supplementary Materials for Reproducibility of Brain Responses: High for Speech Perception, Low for Reading Difficulties [file 41598_2019_41992_MOESM1_ESM.docx]

Supplementary Materials for

**Reproducibility of Brain Responses: High for Speech Perception, Low for Reading Difficulties**

**Main authors:** Paavo H. T. Leppänen^a,^*, Dénes Tóth^b^, Ferenc Honbolygó^b^, Kaisa Lohvansuu^a^, Jarmo A. Hämäläinen^a^, NEURODYS WP7 group†, Jean-Francois Demonet^c,d^, Gerd Schulte-Körne^e^, Valéria Csépe^b^

**Affiliations:**

^a^ Centre for Interdisciplinary Brain Research, Department of Psychology, P.O. Box 35, 40014 University of Jyväskylä, Finland.

^b^ Brain Imaging Centre, Research Centre for Natural Sciences, Hungarian Academy of Sciences, Hungary, 1519 Budapest, P.O. Box 286

^c^ Université de Toulouse, UPS, Imagerie cérébrale et handicaps neurologiques UMR 825; CHU Purpan, Place du Dr Baylac, F-31059 Toulouse Cedex 9, France

^d^ Leenaards Memory Center, Département Neurosciences Cliniques, Centre Hospitalier Universitaire Vaudois (CHUV) & University of Lausanne, Rue du Bugnon 46, CH-1011 Lausanne, Switzerland

^e^ Department of Child and Adolescent Psychiatry, Psychosomatics, and Psychotherapy, Ludwig-Maximilians-Universität, Nußbaumstr. 5a, 80336 Munich, Germany

†**Other authors of** NEURODYS WP7 group: Jürgen Bartling^e^, Jennifer Bruder^e^, Yves Chaix^c^, Stephanie Iannuzzi^f^, Rodolphe Nenert^f^, Nina Neuhoff^e^, Silke Streiftau^e^, Annika Tanskanen^a^, Jyrki Tuomainen^g^

**Affiliations:**

^f^ Inserm, Imagerie cérébrale et handicaps neurologiques UMR 825; F-31059 Toulouse, France

^g^ Language and Cognition, University College London, Gower Street, London WC1E 6BT, UK

*Corresponding author: Paavo H.T. Leppänen

Department of Psychology, P.O.Box 35, 40014 University of Jyväskylä, Finland

Email: paavo.ht.leppanen@jyu.fi

Tel. +358503384747

**This PDF file includes:**

Supplementary results

Figures: Supplementary Figures 1 - 9

**Supplementary Results**

In this section, we present a more detailed view of the statistical effects of the investigated experimental factors. Because the generalized dissimilarity – the main output of the TANOVA analysis – has no inherent scale, we introduced a further transformation step while visualizing the results (see Supplementary Figs. 1, 3, 5, 6): at each time point, the generalized dissimilarity was standardized to z-score based on its respective random distribution. In all these figures, colored sections of the Time axis show uncorrected p-values; colored areas under the effect curve indicate corrected p-values (persistently significant effects). For details, see 28, pp. 176-178.

To aid the interpretation of the two persistent effects – the main effect of stimulus type and the interaction between reading group and stimulus type – we also plotted the scalp topographies of these effects at representative time points (see Supplementary Figs. 2, 4).

**The effect of stimulus type**

Supplementary Figure 1 provides a detailed view of the stimulus type effect in both dyslexic and control groups and speech and non-speech conditions in all national samples. In Supplementary Figure 2, the dynamics of the scalp topographies for the stimulus type effect are shown in 50 ms time steps. The dynamics and magnitude of the statistical effects were highly similar across the national samples, especially for the control groups.

**Reading group effects**

Contrary to the robust effects of the stimulus type (deviant vs. standard) in both the speech and non-speech conditions in all national samples (Fig. 2 and Supplementary Fig. 1), the group differences between dyslexic and control readers were highly sample- and condition-dependent, which is evident from both the standardized effect curves (Supplementary Fig. 3) and the scalp topography distribution maps (Supplementary Fig. 4), as well as from the violin plot maps Supplementary Fig. 7).

**The effect of language exemplars**

The language exemplar factor did not modulate the effect of the two other factors for speech MMN or LDN in any of the national samples: We found neither a three-way interaction between the language exemplar, stimulus type, and reading group effects (Supplementary Fig. 5), nor a two-way interaction between the language exemplar and stimulus type effect (Supplementary Fig. 6 and Supplementary Fig. 9). This shows that there is no systematic processing difference of different exemplars of the same vowel category when phonetically similar (i.e. within category vowels) but acoustically different speech sounds (/y/’s) are contrasted to a different speech sound category (/i/). This suggests that the processing of different exemplars is based on an invariant vowel identity code enabling vowel identification amongst wide acoustic variation (1).

**The violin plots of the MMN, P3a, LDN responses in the control and dyslexic groups in the different national samples**

Supplementary Fig. 7 displays the violin plots of the GFP amplitudes for the MMN, P3a, LDN responses in the control and dyslexic groups in the different national samples showing that group differences in each country are relatively small.

**The signal-to-noise ratio in different national samples**


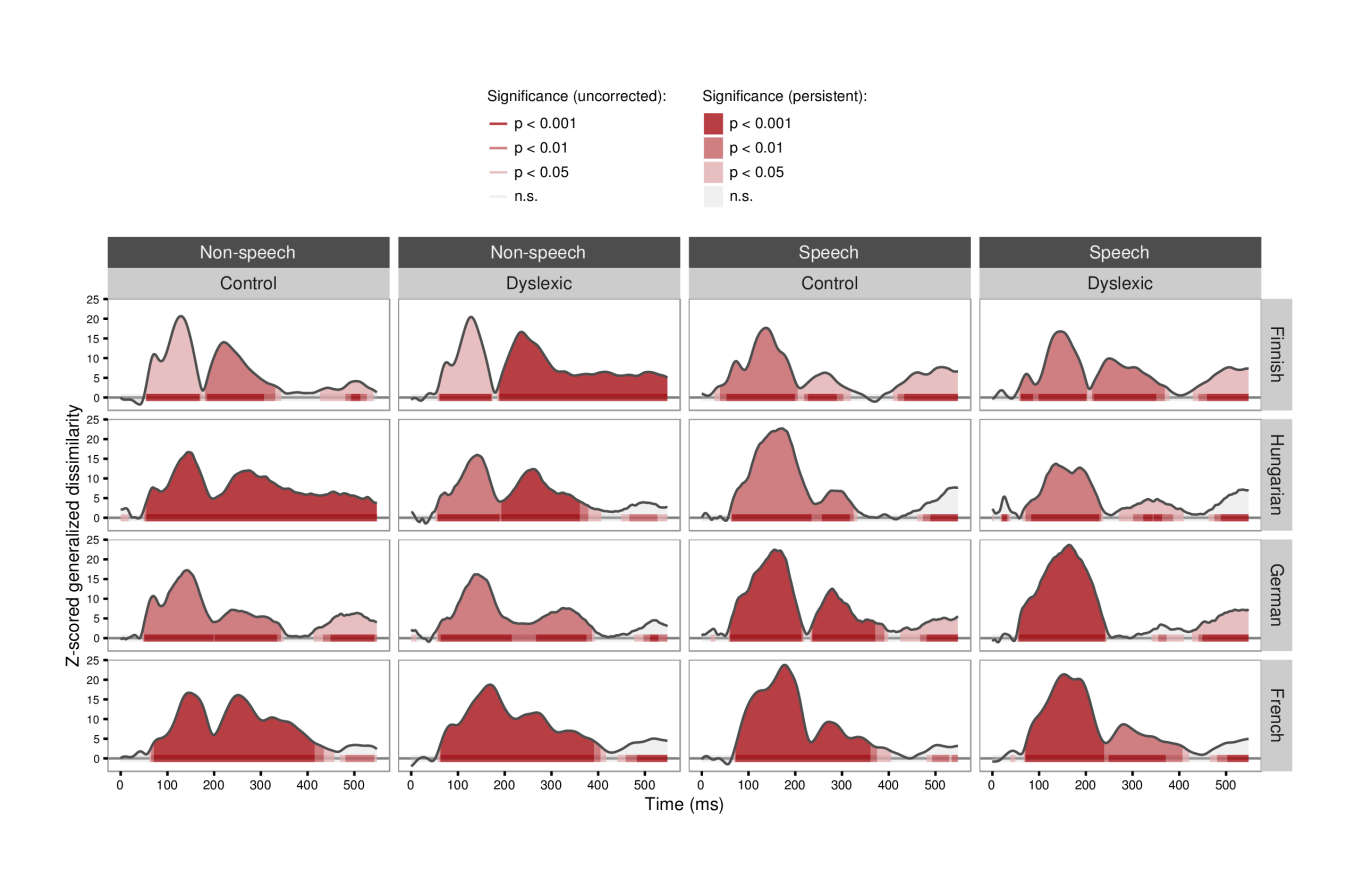
The signal-to-noise ratio (SNR) in different national samples is displayed in Supplementary Fig. 8. The SNR was estimated by first calculating grand average difference waves (ERPs to the deviant minus ERPs to the standard stimuli) for each national sample, reading group, condition, and channel separately. Next, the standard deviation (SD) was calculated for each of these difference waves at -50-0 ms baseline and then the ERP amplitude of the difference waves at each post-stimulus time point was divided by this pre-stimulus SD. Next the averages of these values were calculated for each national sample, reading group and condition. For such an approach, see e.g. (2, 3). The figure shows that, overall, the SNR values are high, at minimum 20 at the MMN time window. This is also confirmed by the very robust and similar results for the MMN response obtained in different laboratories. Further, we could not identify any systematic laboratory or national sample effect pattern on SNR.

**Supplementary Figure 1**

Standardized effects (Z-scored generalized dissimilarities, see Supplementary Text) of stimulus type (deviant vs. standard). The deviant response was the average of the responses to Finnish-Hungarian, German and French /y/s in the non-speech (left panels) and speech (right panels) conditions.


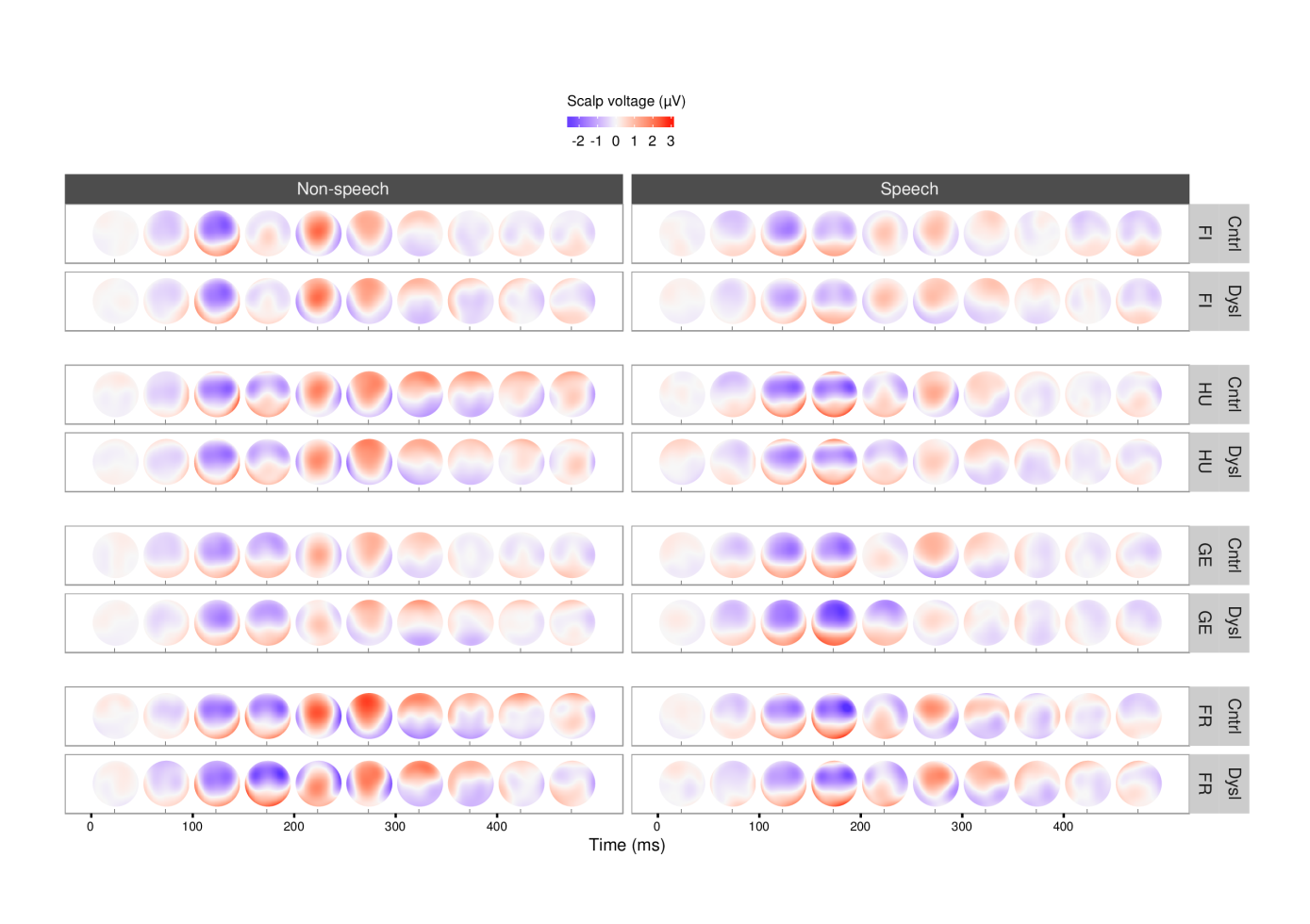
**Supplementary Figure 2**

Scalp topographies of the raw differences between stimulus types (deviant-standard) in control (Cntrl) and dyslexic (Dysl) readers in 50 ms time steps in the non-speech (left panel) and speech conditions (right panel). The topographies and their dynamics were extremely similar across the national samples, especially in the MMN time window (100-200 ms). The deviant response was the average of the responses to Finnish-Hungarian, German and French /y/s. FI = Finland, HU = Hungary, GE = Germany, FR = France.
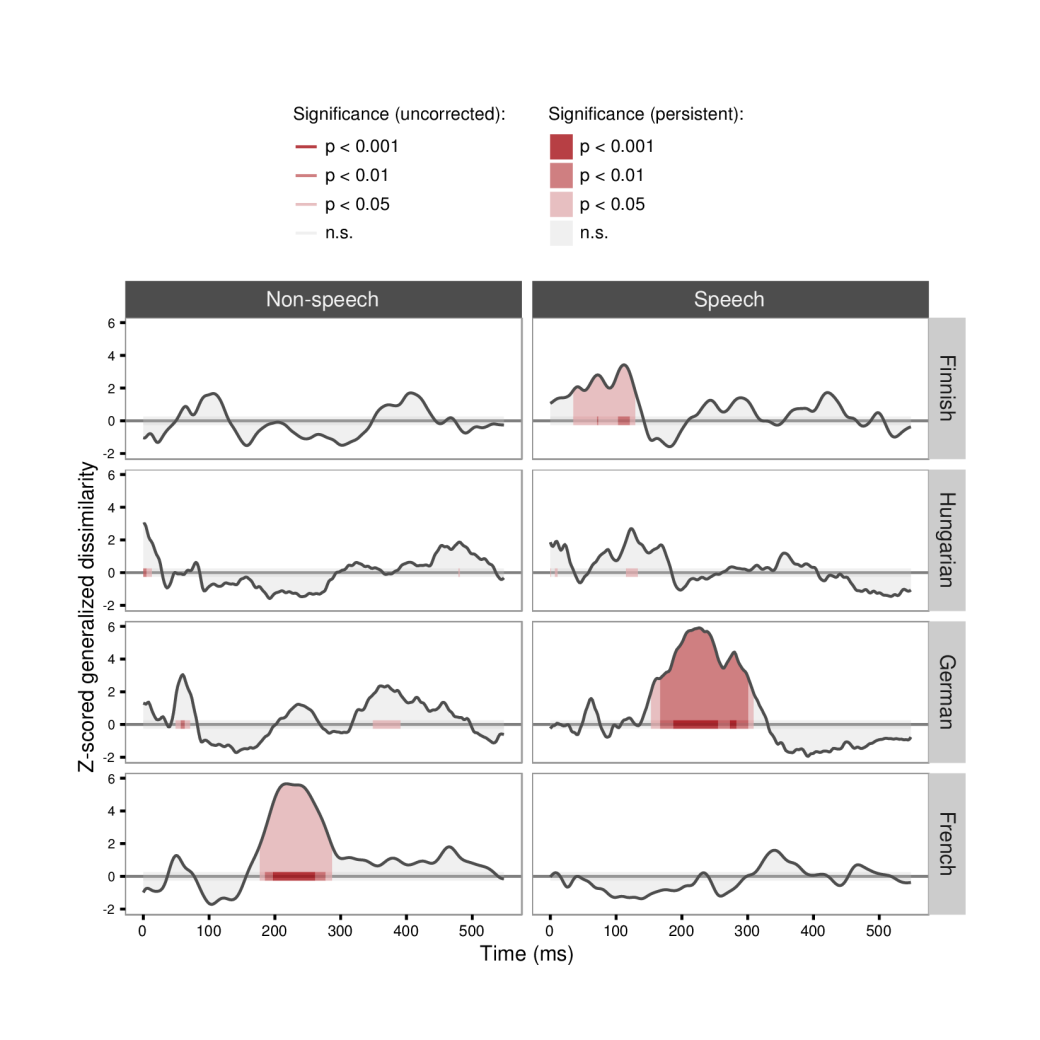
**Supplementary Figure 3**

Time course of the interaction between reading group (Dyslexic vs. Control) and stimulus type (deviant vs. standard; the deviant response was the average of the responses to Finnish-Hungarian, German and French /y/s). Contrary to the main effect of stimulus type (see Supplementary Fig. 1), the magnitude and dynamics of the interaction (that is, the effect of reading group on the deviant-standard difference) showed substantial variation between the national samples in both stimulus categories.


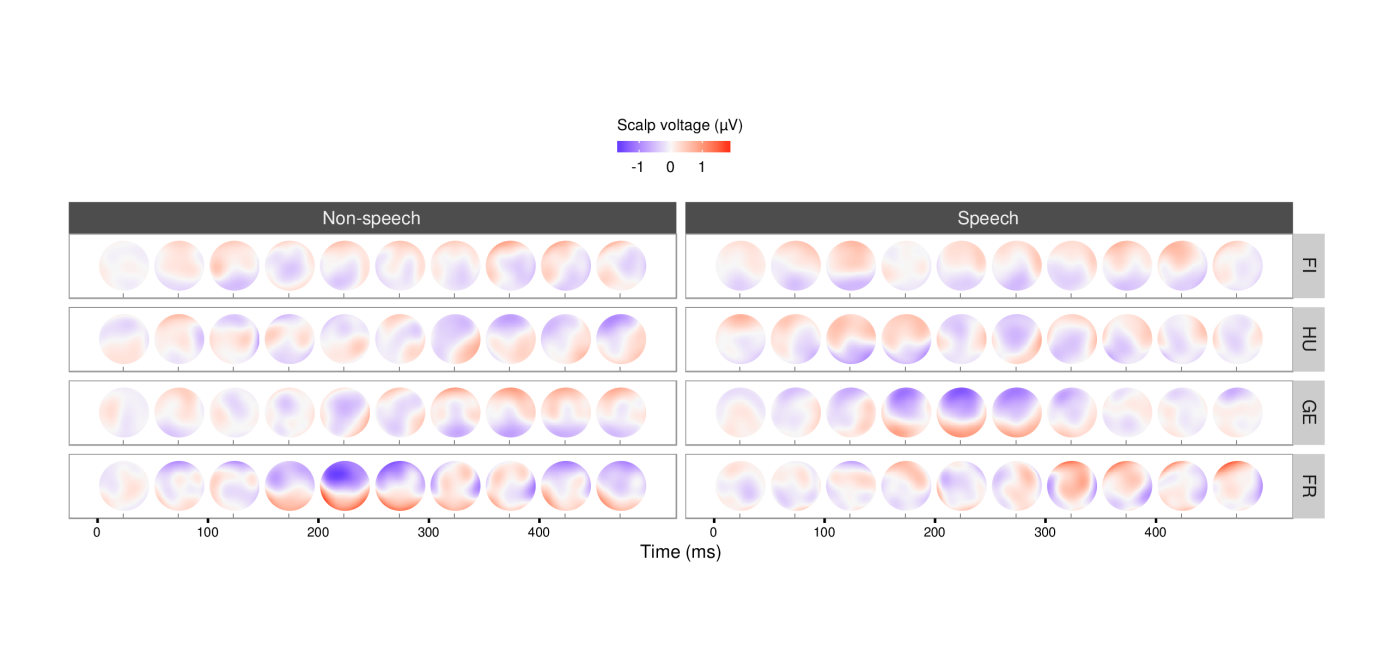
**Supplementary Figure 4**

Scalp topographies of the raw difference between reading groups (Dyslexic-Control) for the deviant-standard difference waves plotted in 50 ms time steps. The reading group effect showed large variation across the national samples. FI = Finland, HU = Hungary, GE = Germany, FR = France.


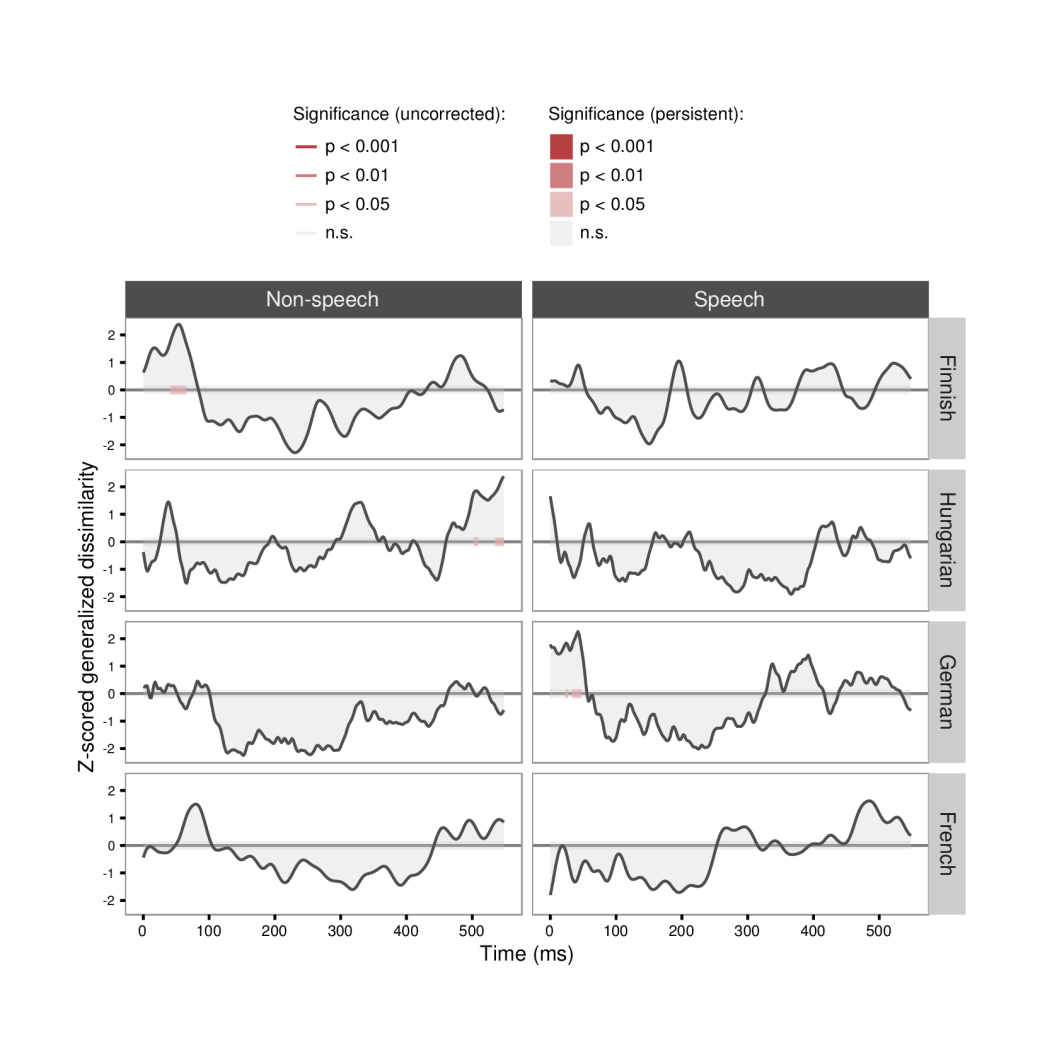


**Supplementary Figure 5**

Time course of the three-way standardized interaction effect of stimulus type (deviant, standard), language exemplar (Finnish-Hungarian, German, French) and reading group (Control, Dyslexic). The language exemplar factor did not modulate the effects of the two other factors in any of the samples (see also Supplementary Fig. 6).


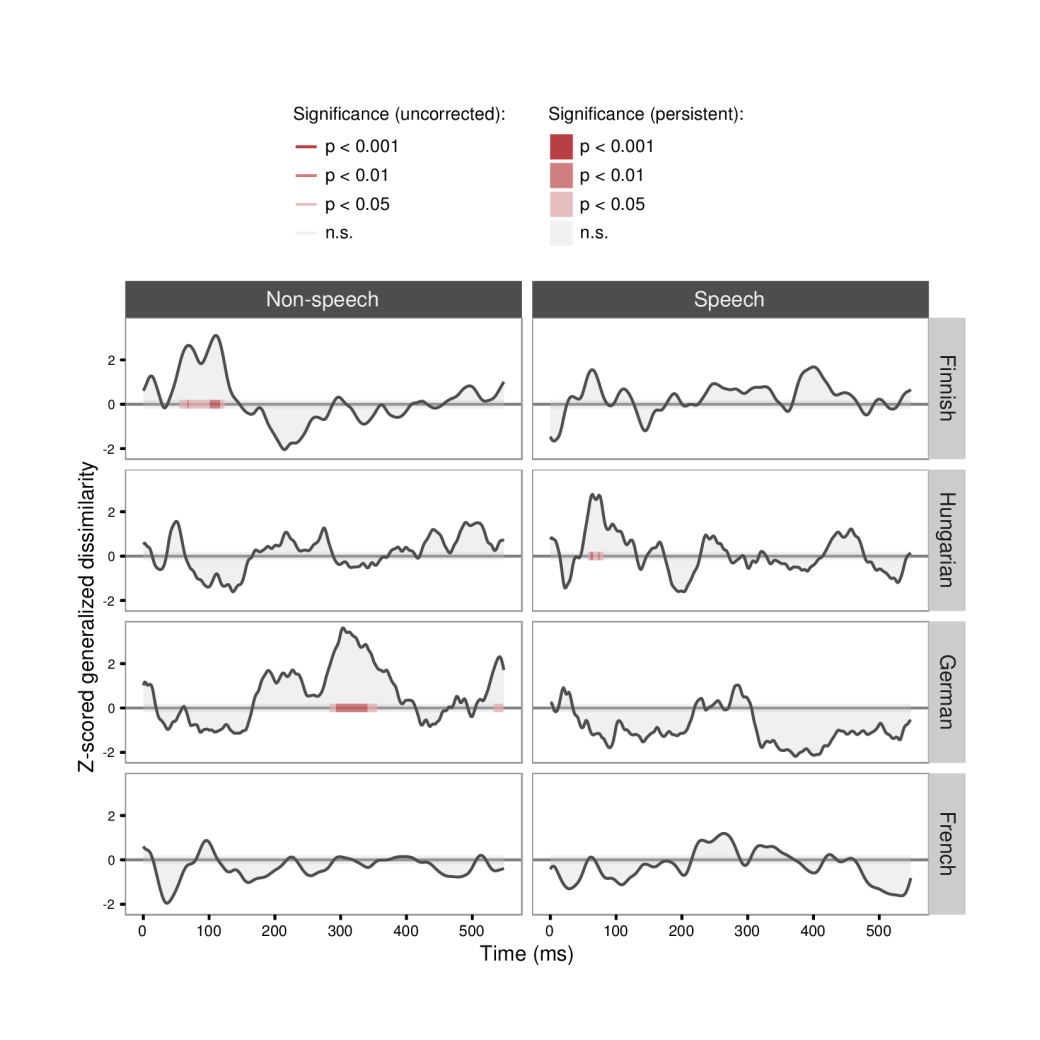
**Supplementary Figure 6**

Time course of the two-way standardized interaction effect of stimulus type (deviant, standard) and language exemplar (Finnish-Hungarian, German, French). The language exemplar effects were ignorable and non-persistent in all four national samples, showing that there were no systematic differences between the processing of acoustically different /y/-vowels.


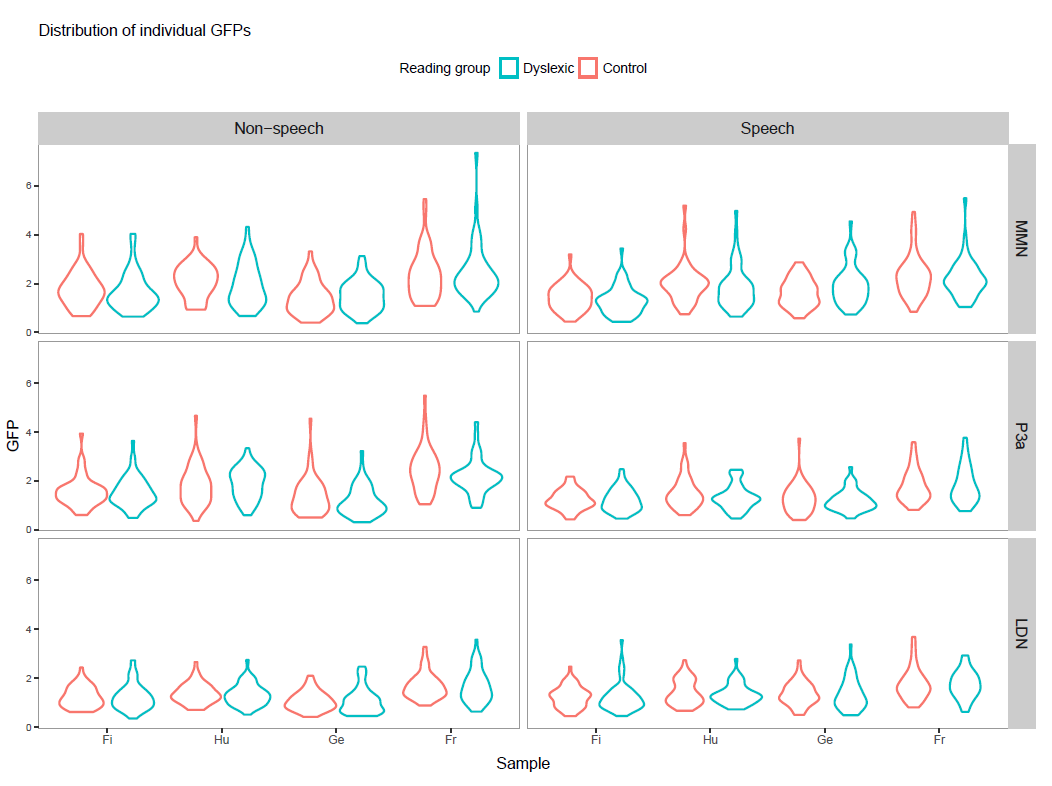
**Supplementary Figure 7**

The violin plots of the MMN, P3a, LDN responses in the control and dyslexic groups in the different national samples. FI = Finland, HU = Hungary, GE = Germany, FR = France.

**
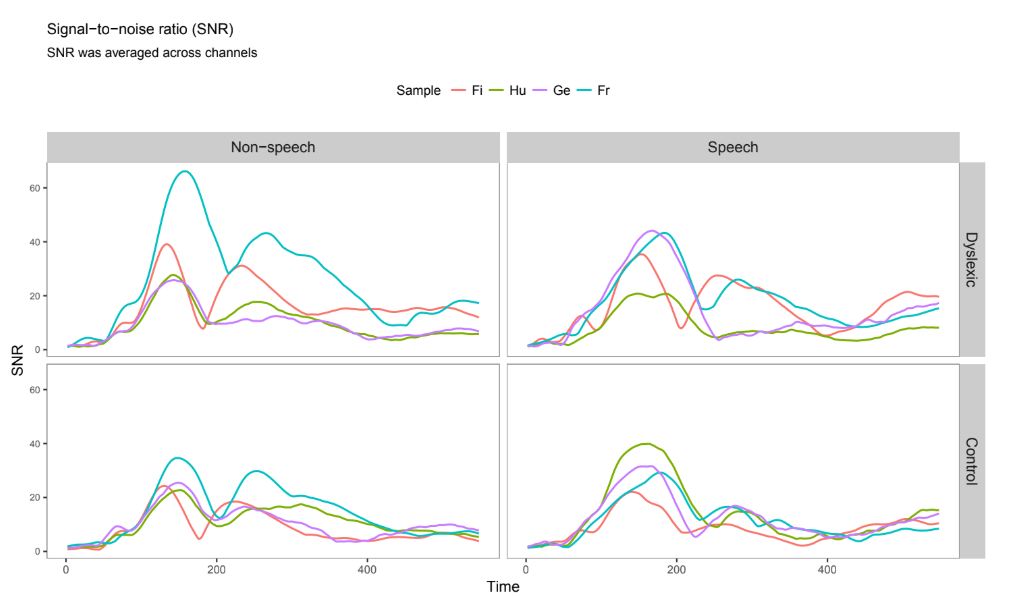
**

**Supplementary Figure 8**

The signal-to-noise ratio (SNR) in different national samples, Fi = Finland, Hu = Hungary, Ge = Germany, Fr = France. SNR was averaged across channels.


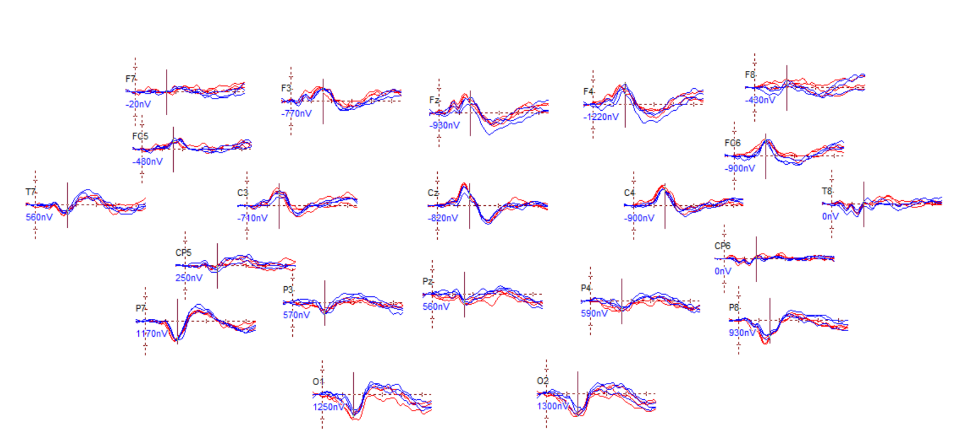


Finnish


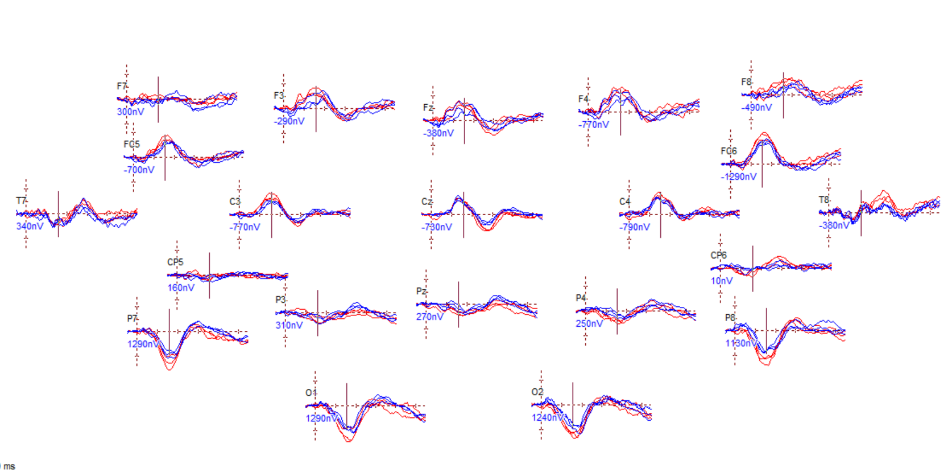


Hungarian


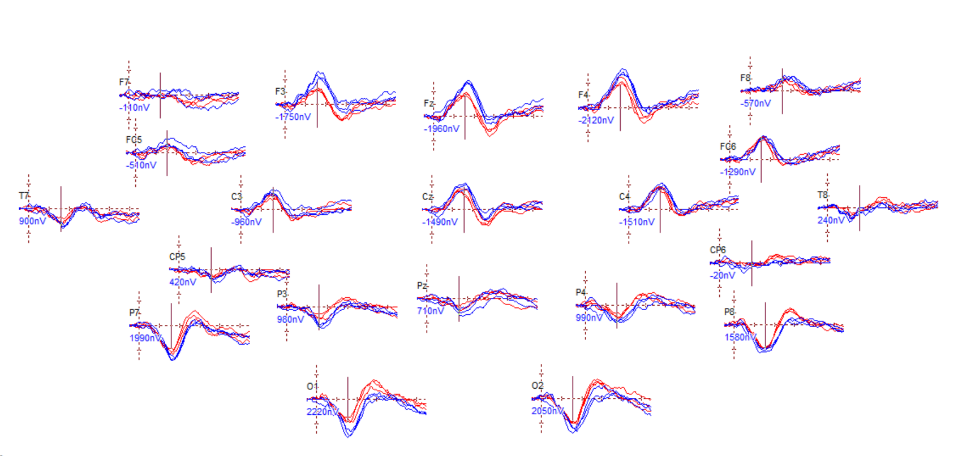


German


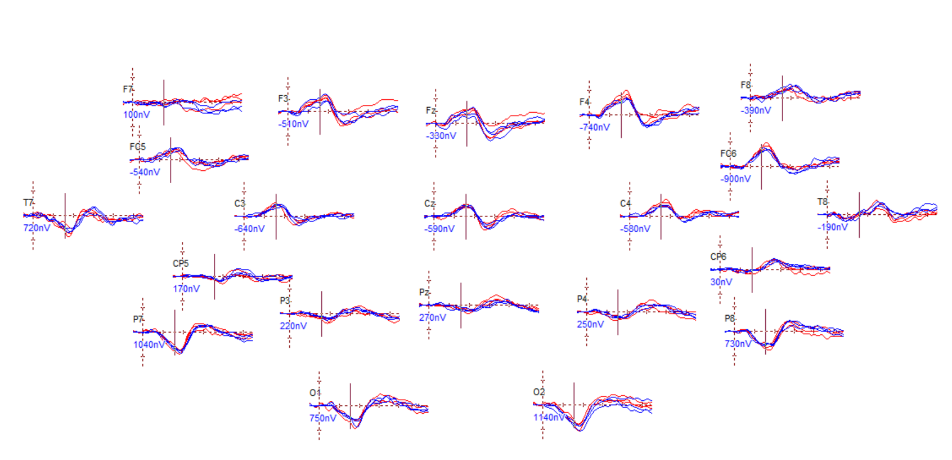


French

**Supplementary Figure 9**

Grand average difference waves (deviant /y/ - standard /i/) from different countries (the language exemplars plotted overlaid). Red lines = control participants, Blue lines = dyslexic participants.

**References for Supplementary materials**

1. Näätänen, R., Paavilainen, P., Rinne, T., Alho, K. The mismatch negativity (MMN) in basic research of central auditory processing: a review. *Clin Neurophysiol*. **118**, 2544–2590 (2007).
2. Debener, S., Strobel, A., Sorger, B., Peters, J., Kranczioch, C., Engel, A. K., Goebel, R. Improved quality of auditory event-related potentials recorded simultaneously with 3-T fMRI: removal of the ballistocardiogram artefact. *NeuroImage*, **34**, 587-597 (2007).
3. Spencer, K. M. Averaging, detection, and classification of single-trial ERPs. In T.C. Handy (Ed.), *Event-related potentials: a methods handbook* (MIT Press, Cambridge, Mass., 2005), pp. 209-227.
